# Study design and rationale for a randomised, placebo-controlled, double-blind study to assess the efficacy of selumetinib (AZD6244; ARRY-142886) in combination with dacarbazine in patients with metastatic uveal melanoma (SUMIT)

**DOI:** 10.1186/s12885-015-1470-z

**Published:** 2015-06-10

**Authors:** Richard D. Carvajal, Gary K. Schwartz, Helen Mann, Ian Smith, Paul D. Nathan

**Affiliations:** 1 Division of Hematology/Oncology, Columbia University Medical Center, New York, NY 10032 USA; 2 AstraZeneca, Macclesfield, UK; 3 Mt Vernon Cancer Centre, Northwood, UK

**Keywords:** Dacarbazine, GNAQ, GNA11, Metastatic uveal melanoma, Phase III, Selumetinib

## Abstract

**Background:**

Uveal melanoma is characterised by mutations in *GNAQ* and *GNA11*, resulting in Ras/Raf/MEK/ERK pathway activation. Treatment with selumetinib (AZD6244, ARRY-142886), a MEK1/2 inhibitor, results in antitumour effects in uveal melanoma pre-clinical models. A randomised phase II trial demonstrated improved progression-free survival (PFS) and response rate (RR) with selumetinib monotherapy versus chemotherapy with temozolomide or dacarbazine in patients with metastatic uveal melanoma. Pre-clinically, selumetinib in combination with alkylating agents enhanced antitumour activity compared with chemotherapy alone. We hypothesise that selumetinib in combination with dacarbazine will result in improved clinical outcomes in patients with metastatic uveal melanoma versus dacarbazine alone.

**Methods/Design:**

SUMIT is a randomised, international, double-blind, placebo-controlled, phase III study assessing the efficacy and safety of selumetinib in combination with dacarbazine in patients with metastatic uveal melanoma who have not received prior systemic therapy. Primary endpoint is PFS. Secondary endpoints include objective RR, duration of response, change in tumour size at Week 6, overall survival, safety and tolerability. Exploratory endpoints include efficacy in tumours with *GNAQ* or *GNA11* mutations. Eligible patients must have: ≥1 lesion that can be accurately measured at baseline, and is suitable for accurate repeated measurements; ECOG performance status 0–1; life expectancy >12 weeks. Mutation status for *GNAQ*/*GNA11* will be assessed retrospectively.

An estimated 128 patients from approximately 50 sites globally will be randomised (3:1) to selumetinib 75 mg twice daily or placebo in combination with dacarbazine 1000 mg/m^2^ on Day 1 of every 21-day cycle until objective disease progression, intolerable toxicity or occurrence of another discontinuation criterion. Randomisation will be stratified by the presence/absence of liver metastases. Tumours will be evaluated by RECIST v1.1 every 6 weeks. All patients have the option of receiving selumetinib with or without dacarbazine at disease progression. Study enrolment began in April 2014 and is expected to complete in early 2015.

**Discussion:**

Treatment of patients with metastatic uveal melanoma represents an area of high unmet medical need. This study evaluating selumetinib in combination with dacarbazine was designed with input from the US FDA, and is the first potential registration trial to be conducted in patients with metastatic uveal melanoma.

**Trial registration:**

Clinicaltrials.gov (Date of registration, October 10, 2013)

Registration number: NCT01974752

Trial abbreviation: SUMIT

## Background

Uveal melanoma is the most common primary tumour of the eye [1]. Biologically distinct from cutaneous melanoma, it is a rare disease with an incidence per year of about 1200–1500 new cases in the US, accounting for around 5 % of all melanomas, and approximately 460 cases in Europe [2–4]. Metastasis is common, occurring in approximately 50 % of patients with posterior uveal melanoma within 15 years of the initial diagnosis and treatment [5], and prognosis is poor with a median overall survival (OS) of 4–15 months [6, 7].

Agents with regulatory approval for use in patients with advanced cutaneous melanoma have only a limited role in the treatment of advanced uveal melanoma, and there are no approved or effective therapies for the treatment of patients with this disease [6]. Although immunotherapy with ipilimumab has been demonstrated to improve survival in patients with metastatic cutaneous melanoma [8] (**NCCN Practice Guidelines in Oncology melanoma version 4.2014** [http://www.nccn.org/professionals/physician_gls/f_guidelines.asp#melanoma]), the efficacy of this agent in uveal melanoma is not well defined. Analysis of single- and multi-centre expanded access programmes indicates modest radiographic response rates in patients with metastatic uveal melanoma; however, any effect upon overall survival has yet to be demonstrated [9–14]. Some benefit has been observed with high dose interleukin-2, another immunological agent to be approved for the treatment of metastatic melanoma, in this patient population [15, 16]. Further prospective data are required to fully understand the potential value of immunotherapy, including pembrolizumab which was recently approved in the US, in this setting.

Vemurafenib and dabrafenib are small molecule inhibitors of *BRAF* approved for use in patients with advanced melanoma harbouring a V600 *BRAF* mutation (**NCCN Practice Guidelines in Oncology melanoma version 4.2014** [www.nccn.org/professionals/physician_gls/pdf/melanoma.pdf]). Antitumour efficacy is only observed in cells harbouring a *BRAF* mutation, with paradoxical activation of the Ras/Raf/MEK/ERK pathway observed in cells with wild-type *BRAF* [17–19]. Given that *BRAF* mutations are absent or rare in uveal melanoma [20–22], there is no utility for these agents in this disease.

Importantly, 80–96 % of uveal melanomas harbour mutations in either the guanidine nucleotide binding protein (G protein), Q polypeptide 1 (*GNAQ*) or the G protein alpha 11 (*GNA11*) gene, in a mutually exclusive pattern [23–25]. Oncogenic mutations in *GNAQ* and *GNA11* result in constitutive activation of these proteins and downstream signalling of pathways such as the YAP pathway [26, 27], the phosphoinositide-3 kinase/AKT [28] and the Ras/Raf/MEK/ERK pathway, thus playing a key role in the development and progression of uveal melanomas [23, 24, 29]. This biology suggests that inhibition of one or more of these signalling pathways may result in antitumour activity.

Selumetinib (AZD6244; ARRY-142886) is an orally available, potent and selective, non-ATP-competitive mitogen-activated protein kinase (MEK1/2) inhibitor [30]. In pre-clinical tumour models, selumetinib demonstrates single agent anti-cancer activity [31], including in models of uveal melanoma harbouring *GNAQ* or *GNA11* mutations [32, 33].

In a hypothesis-generating phase II open-label study, patients with metastatic uveal melanoma, who were either temozolomide or dacarbazine treatment naïve, achieved an improved progression-free survival (PFS) with selumetinib versus chemotherapy alone with temozolomide or dacarbazine (15.9 vs 7 weeks; hazard ratio [HR] 0.46 [95 % confidence interval (CI) 0.30, 0.71]; p < 0.001). Tumour regression was observed in 49 % of patients treated with selumetinib. No Response Evaluation Criteria In Solid Tumors (RECIST) responses were observed in patients treated with chemotherapy [34].

Pre-clinical work has identified several promising strategies to improve the efficacy achieved with selumetinib alone, including the concurrent administration of chemotherapy with selumetinib, which results in increased expression of pro-apoptotic proteins such as BIM [35]. When evaluated in combination with chemotherapy, selumetinib enhanced antitumour efficacy compared with each agent alone, with particular sensitivity to *BRAF/RAS*-mutant tumours [31]. Selumetinib in combination with temozolomide, which has the same active metabolite as dacarbazine, enhanced tumour growth inhibition, DNA damage and apoptosis in a *RAS*-mutant tumour model versus temozolomide monotherapy [35]. A series of clinical trials assessing the efficacy of selumetinib in combination with chemotherapy have shown promise in patients with mutations associated with the *KRAS* and *BRAF* pathways [36, 37], including in combination with dacarbazine in patients with *BRAF* mutation-positive cutaneous or unknown melanoma [37].

This pre-clinical [31, 35] and clinical evidence [34, 36, 37] suggests that targeting the Ras/Raf/MEK/ERK pathway in combination with chemotherapy is an attractive therapy option to investigate in this disease setting. We therefore hypothesise that selumetinib in combination with dacarbazine, an alkylating agent approved for use in the treatment of advanced melanoma [38] (**NCCN Practice Guidelines in Oncology melanoma version 4.2014** [www.nccn.org/professionals/physician_gls/pdf/melanoma.pdf]), may offer improved clinical outcomes in patients with metastatic uveal melanoma versus dacarbazine alone.

## Methods/Design

### Study objectives

The primary objective is to assess the efficacy of selumetinib in combination with dacarbazine compared with placebo in combination with dacarbazine in terms of PFS in patients with metastatic uveal melanoma (Table 1). Assessment will be by blinded independent central review (BICR) of computed tomography (CT) or magnetic resonance imaging (MRI) scans according to RECIST v1.1.

**Table 1:** Key study objectives

Primary objective	Exploratory objectives
• Progression-free survival	• Overall survival adjusting for the impact of treatment options available post-progression
Secondary objectives	• Symptoms and HRQoL using the EORTC-QLQC30 v3
• Objective response rate	• Hospital-related resource use and health state utility
• Duration of response	• Pharmacokinetics versus clinical outcomes, efficacy, AEs and/or safety parameters
• Change in tumour size at Week 6	• Explore MEK pathway mutations in *GNAQ* and *GNA11*
• Overall survival	• Biomarkers for response or development of cancer
• Safety and tolerability profile	• Host genetic polymorphisms

*AE* adverse event, *EORTC-QLQC30 v3* European Organisation for Research and Treatment of Cancer 30-item core quality of life questionnaire version 3, *GNAQ* guanidine nucleotide binding protein (G protein), Q polypeptide 1, *GNA11* G protein alpha 11, *HRQoL* health-related quality of life

Secondary objectives include further assessment of efficacy in terms of OS and objective response rate (ORR), duration of response (DoR), change in tumour size at Week 6, safety and tolerability. Exploratory objectives include assessment of mutations in *GNAQ/GNA11*, health-related quality of life (HRQoL) and biomarkers for response or development of cancer.

### Trial design and treatment plan

**Fig. 1:**
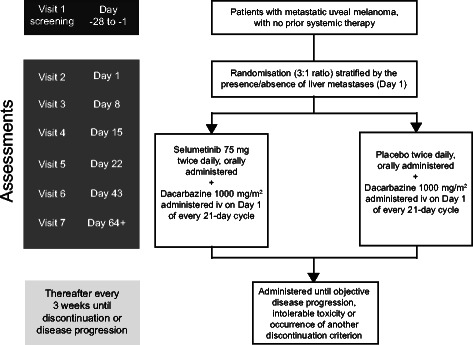
Study design. iv = intravenous

SUMIT (NCT01974752) is a randomised, international, double-blind, placebo-controlled phase III study assessing the efficacy and safety of selumetinib (75 mg, twice daily on a continuous oral administration) in combination with dacarbazine (1000 mg/m^2^, intravenously on Day 1 of every 21-day cycle) compared with matched placebo in combination with dacarbazine (same schedule) in patients who have not previously had a systemic therapy for metastatic uveal melanoma (Fig. 1).

Patients will be randomised in a 3:1 ratio to receive selumetinib in combination with dacarbazine or placebo in combination with dacarbazine, and stratified by the presence/ absence of liver metastases (yes/no) at randomisation. Following confirmation of objective disease progression by BICR, all patients have the option of receiving open-label selumetinib with or without dacarbazine or an alternative treatment approach.

All randomised patients will be assessed by CT or MRI at screening, Week 6 and every 6 weeks thereafter, relative to the date of randomisation until objective disease progression regardless of whether or not they are on study treatment. Up to the data cut-off for the primary analysis, RECIST v1.1, defined by BICR, will be used to assess each patient’s tumour response to treatment and allow calculations of PFS, ORR, duration of response and tumour size at Week 6. For patients receiving open-label selumetinib with or without dacarbazine, tumour assessments will be performed in accordance with local practice at the investigational site and will not be sent for BICR.

Adverse events (AEs) will be collected from the time of informed consent, coded using the Medical Dictionary for Regulatory Activities (MedDRA), and graded using the National Cancer Institute Common Terminology Criteria for Adverse Events (CTCAE). AEs will continue to be collected for patients who opt to receive open-label selumetinib (either alone or in combination with dacarbazine) as post-progression therapy.

Ophthalmologic examinations and echocardiogram/multi-gated acquisitions will be performed at randomisation and then every 6 and 12 weeks, respectively, thereafter or as clinically indicated. For both, a 30-day follow-up assessment will be required if an on-treatment assessment was abnormal at the time of discontinuation of selumetinib/placebo, to confirm reversibility of the abnormality.

The European Organization for Research and Treatment of Cancer 30-item core quality of life questionnaire version 3 will be used to assess HRQoL at baseline and thereafter every 3 weeks following randomisation until objective disease progression or death.

Archival tumour samples will be collected for all randomised patients for the assessment of *GNAQ/GNA11* mutation status. In addition, patients will provide plasma samples for analysis of circulating free tumour DNA. Correlation between tumour and plasma-based mutation analysis will be assessed.

All patients are required to provide written informed consent. The study will be performed in accordance with the Declaration of Helsinki and the International Conference on Harmonisation Good Clinical Practice. The protocol was approved by the Institutional Review Board at each study site (approximately 50 sites, Table 2) and complied with local country regulations.

**Table 2:** Ethics committees

Research site	Ethics committee
USA	
Los Angeles, CA	University of California Los Angeles, Institutional Review Board
Aurora, CO	Western Institutional Review Board
Miami Beach, FL	Mount Sinai Medical Center, Institutional Review Board
Atlanta, GA	Emory University, Institutional Review Board
Lutherville, MD	John Hopkins Medicine, Office of Human Subjects Research, Institutional Review Board
Rochester, MN	Mayo Clinic, Institutional Review Board
St Louis, MO	Washington University in St Louis, Human Research Protection Office
Morristown, NJ	Atlantic Health System, Institutional Review Board
New York, NY	Columbia University Medical Center, Institutional Review Board
New York, NY	Memorial Sloan-Kettering Cancer Center, Institutional Review Board
Chapel Hill, NC	The University of North Carolina at Chapel Hill, Office of Human Research Ethics
Philadelphia, PA	Jefferson, Office of Human Research Ethics, Institutional Review Board
Charleston, SC	Western Institutional Review Board
Charlottesville, VA	University of Virginia, Institutional Review Board
Belgium	
Edegem	Ethisch Comite Unversitair Ziekenhuis Antwerpen
Gent	Ethisch Comite Unversitair Ziekenhuis Antwerpen
Kortrijk	Ethisch Comite Unversitair Ziekenhuis Antwerpen
Leuven	Ethisch Comite Unversitair Ziekenhuis Antwerpen
Canada	
Toronto, ON	University Health Network Research Ethics Board
Montreal, QC	Comité d’éthique de la recherche du CHUM
Czech Republic	
Hradec Kralove	Eticka komise Fakultni nemocnice Hradec Kralove
Olomouc	Eticka komise Fakultni nemocnice Olomouc a Lekarske UP v Olomouci
Prague	Eticka komise Pri Institutu Klinicke a experimentalni Mediciny a Thomayerove Nemocnici
Finland	
Hus	Varsinais-Suomen sairaanhoitopiirin kuntayhtyma
Tampere	Varsinais-Suomen sairaanhoitopiirin kuntayhtyma
France	
Nice	Groupe Hospitalier Pitie-Salpetriere
Paris	Groupe Hospitalier Pitie-Salpetriere
Germany	
Heidelberg	Ethikkommission Bei Der LMU Munchen
Munich	Ethikkommission Bei Der LMU Munchen
Israel	
Jerusalem	Ethics Helsinki Committee at Hadassah University Hospital
Ramat Gan	Helsinki Committee Clinical Trials Approval Committee Tel Hashomer Medical Center
Netherlands	
Leiden	Commissie Medische Ethiek H1-Q
Spain	
Barcelona	Hospital Universitario ramon y cajal Clinical Research Ethics Committee
Barcelona	Hospital Universitario ramon y cajal Clinical Research Ethics Committee
L'Hospitalet de Llobregat	Hospital Universitario Ramon y Cajal Clinical Research Ethics Committee
Madrid	Hospital Universitario Ramon y Cajal Clinical Research Ethics Committee
Madrid	Hospital Universitario Ramon y Cajal Clinical Research Ethics Committee
Seville	Hospital Universitario Ramon y Cajal Clinical Research Ethics Committee
Valencia	Hospital Universitario Ramon y Cajal Clinical Research Ethics Committee
UK	
Birmingham	NRES Committee London
Glasgow	NRES Committee London
Northwood	NRES Committee London
Nottingham	NRES Committee London
Southampton	NRES Committee London
Swansea	NRES Committee London

### Study population

Patients will be eligible for inclusion if they are ≥18 years of age with a clinical diagnosis of metastatic uveal melanoma (histologically or cytologically confirmed), and have ≥1 lesion that can be accurately measured at baseline, and is suitable for accurate repeated measurements. Patients must have an Eastern Cooperative Oncology Group (ECOG) performance status 0–1, a life expectancy >12 weeks and be able to provide informed consent.

Patients will be excluded from the study if they have received previous treatment with a systemic anticancer therapy, or have symptomatic brain metastases or spinal cord compression. Full patient selection criteria are presented in Table 3.

**Table 3:** Key patient selection criteria

Inclusion criteria	Exclusion criteria
• Clinical diagnosis of metastatic uveal melanoma	• Any prior systemic anticancer therapy, including for the treatment of this current diagnosis
• Histological or cytological confirmation of melanoma	• An investigational drug within 30 days of starting treatment, or has not recovered from side effects of an investigational drug
• Male or female aged ≥18 years	
• Suitable for treatment with dacarbazine chemotherapy	• Any non-systemic anticancer therapy that has not been cleared from the body by the time of starting study treatment
• ≥1 lesion that can be accurately measured at baseline as ≥10 mm in the longest diameter, which is suitable for accurate repeated measurements	
	• Radiation therapy within 4 weeks prior to starting study treatment
	• Major surgery within 4 weeks prior to entry into the study that would prevent administration of study treatment
• ECOG performance status 0–1	
• Life expectancy >12 weeks	
• Normal organ and marrow function	• Any prior investigational therapy comprising inhibitors of RAS, RAF or MEK at any time
• Negative urinary or serum pregnancy test for women with childbearing potential	
	• Previous treatment with dacarbazine
• Able to swallow selumetinib/placebo capsules	• Any unresolved toxicity > CTCAE grade 2 from previous anticancer therapy, excluding alopecia
• Signed informed consent document	
	• History of allergic reactions attributed to compounds of similar chemical or biologic composition to selumetinib or dacarbazine
	• Symptomatic brain metastases or spinal cord compression
	• Cardiac conditions, such as uncontrolled hypertension, acute coronary syndrome, uncontrolled angina or heart failure
	• Severe concomitant systemic disorder, active infection, active bleeding diatheses or renal transplant
	• Refractory nausea and vomiting, chronic gastrointestinal diseases or significant bowel resection that would preclude adequate absorption
	• History of another primary malignancy within 5 years prior to starting study treatment
	• Current or past history of retinal pigmented epithelial detachment/central serous retinopathy; retinal vein occlusion; intraocular pressure >21 mmHg; uncontrolled glaucoma
	• Female patients who are breast-feeding and male or female patients of reproductive potential who are not employing an effective method of birth control
	• Judgement by the investigator that the patient should not participate in the study

*CTCAE* Common Terminology Criteria for Adverse Events, *ECOG* Eastern Cooperative Oncology Group

### Statistical methods

An estimated 128 patients with metastatic uveal melanoma will be randomised 3:1 to the selumetinib plus dacarbazine group (96 patients) or placebo plus dacarbazine group (32 patients), to obtain approximately 93 PFS events. The sample size is driven by the number of required events. Assuming a true PFS HR of 0.46 [34], this number of events will provide 90 % power to demonstrate a statistically significant difference for PFS at a 5 % 2-sided significance level. OS will be analysed at the time of PFS analysis and updated at 65 % maturity (approximately 83 events). Assuming a true OS HR of 0.49, with 83 deaths, the trial has 80 % power to demonstrate a statistically significant difference for OS with a 1-sided type-1 error of 2.43 %. The type-1 error has been adjusted to allow for a single interim analysis based on approximately 45 death events.

Efficacy analyses will be performed on the efficacy analysis set on an intent-to-treat (ITT) basis according to randomised treatment. PFS, based on BICR, and OS will be analysed by a stratified log-rank test, with the presence of liver metastases at randomisation included as a stratification factor. The effect of treatment will be estimated by the HR together with its corresponding 2-sided CI and p-value. Kaplan-Meier plots of PFS and OS will also be presented. ORR (based on BICR) will be analysed using a logistic regression adjusted for the stratification factor presence/absence of liver metastases.

To describe the nature of benefits of selumetinib treatment, PFS, ORR and OS will be tested at a 2-sided significance level of 5 (PFS and ORR based on BICR). In order to strongly control the type-1 error at 2.5 % 1-sided, a multiple testing procedure with an alpha-exhaustive recycling strategy [39] will also be employed across the primary endpoint PFS and secondary endpoints ORR and OS. No formal statistical testing will be performed on the safety data. AEs will be summarised by preferred term and system organ class (using MedDRA). Summaries of AEs by causality and National Cancer Institute CTCAE grade will also be presented.

Data for exploratory patient-reported outcome endpoints will be analysed descriptively for the efficacy (ITT) analysis set. Data will be presented in terms of minimum, maximum, mean, standard deviation and median scores together with 95 % CIs at each visit as well as change from baseline to each scheduled visit (including end of treatment).

## Discussion

We hypothesise that selumetinib in combination with dacarbazine will provide improved clinical outcomes versus dacarbazine alone in patients with metastatic uveal melanoma. This is founded on the encouraging results from a prior phase II study, which reported a statistically significant improvement in PFS for patients with metastatic uveal melanoma receiving selumetinib compared with those receiving chemotherapy (HR 0.46; 95 % CI 0.30, 0.71; p < 0.001) [34]. A comparable PFS improvement to that in the overall population was observed in patients with tumours harbouring a mutation in *GNAQ* or *GNA11* [34].

This phase III trial will build on pre-clinical and clinical evidence assessing the efficacy of selumetinib in combination with chemotherapy. Pre-clinically, the combination of selumetinib with chemotherapy has been shown to increase the cytotoxicity of chemotherapy alone [31], including in *RAS*-mutant tumour models, i.e. cells dependent on the Ras/Raf/MEK/ERK pathway [35]. Clinically, two phase II trials have demonstrated the efficacy of selumetinib in combination with chemotherapy in patients with Ras/Raf/MEK/ERK-pathway-dependent cancer [36, 37]. As noted, tumours dependent on the Ras/Raf/MEK/ERK pathway include uveal melanomas harbouring oncogenic *GNAQ/GNA11* mutations [24]. Thus, the combination of selumetinib with chemotherapy in this disease setting may pose a favourable treatment approach.

Dacarbazine was selected as the therapy for use in combination with selumetinib in this study based on a number of factors. Selumetinib in combination with temozolomide (which has the same active metabolite as dacarbazine but does not require liver metabolism for activation [40]) enhanced the antitumour effect of temozolomide monotherapy [35]. Although this study utilised a human colorectal tumour xenograft model, it provides positive evidence for the efficacy of selumetinib in combination with temozolomide/dacarbazine in a *RAS*-mutant model. The combination of selumetinib and dacarbazine has demonstrated clinical activity in a phase II trial, with significant improvements in PFS observed in patients with *BRAF* mutation-positive advanced cutaneous or unknown melanoma receiving the combination versus dacarbazine alone [37]. Clinically, dacarbazine is the only chemotherapy approved for use in the treatment of melanoma (**NCCN Practice Guidelines in Oncology melanoma version 4.2014** [www.nccn.org/professionals/physician_gls/pdf/melanoma.pdf]) and is the most commonly prescribed chemotherapy for both metastatic cutaneous and uveal melanoma. Taken together, these data provide the rationale for selecting dacarbazine as the combination agent for selumetinib in this study.

To address concerns that dacarbazine may be a less optimal therapy than selumetinib for patients with uveal melanoma, based on the encouraging clinical efficacy with selumetinib in this patient population [34], an unequal randomisation ratio (3:1) will be used in this study for selumetinib and dacarbazine. The evaluation of response at Week 6 and every 6 weeks thereafter will enable the identification of early progressors on chemotherapy and permit rapid crossover of patients to selumetinib, if required. At the point of objective disease progression, patients will have the option of receiving open-label selumetinib with or without dacarbazine. In the phase II study, 86 % of patients with metastatic uveal melanoma were clinically sufficiently fit to receive selumetinib treatment after experiencing disease progression with temozolomide or dacarbazine. In these patients, efficacy with selumetinib was lower with a median PFS of 8 weeks (95 % CI 8, 12 weeks) compared with 15.9 weeks (95 % CI 8.4, 21.1 weeks) when selumetinib was given initially [34]. However, these findings need to be interpreted with caution as this was a post-hoc analysis, and the reasons behind these findings are not clear.

As the treatment of patients with metastatic uveal melanoma represents an area of high unmet medical need, the results of the described phase II studies, coupled with pre-clinical and clinical evidence, provide the rationale for assessing selumetinib in combination with dacarbazine in this patient population. This study is the first potential registration trial to be conducted in patients with metastatic uveal melanoma and was designed with input from the US Food and Drug Administration (FDA). Study enrolment began in April 2014 and the study is expected to complete in early 2015.

## Abbreviations

AE: Adverse event
BICR: Blinded independent central review
BID: Twice daily
CI: Confidence interval
CT: Computed tomography
CTCAE: Common Terminology Criteria for Adverse Events
DoR: Duration of response
ECOG: Eastern Cooperative Oncology Group
EORTC-QLQC30 v3: European Organisation for Research and Treatment of Cancer 30-item core quality of life questionnaire version 3
FDA: Food and Drug Administration
G protein: Guanidine nucleotide binding protein
*GNA11*: Guanidine nucleotide binding protein alpha 11
*GNAQ*: Guanidine nucleotide binding protein, Q polypeptide 1
HR: Hazard ratio
HRQoL: Health-related quality of life
ITT: Intent-to-treat
iv: Intravenous
MedDRA: Medical Dictionary for Regulatory Activities
MEK: Mitogen-activated protein kinase
MRI: Magnetic resonance imaging
ORR: Objective response rate
OS: Overall survival
PFS: Progression-free survival
RECIST: Response Evaluation Criteria In Solid Tumors
